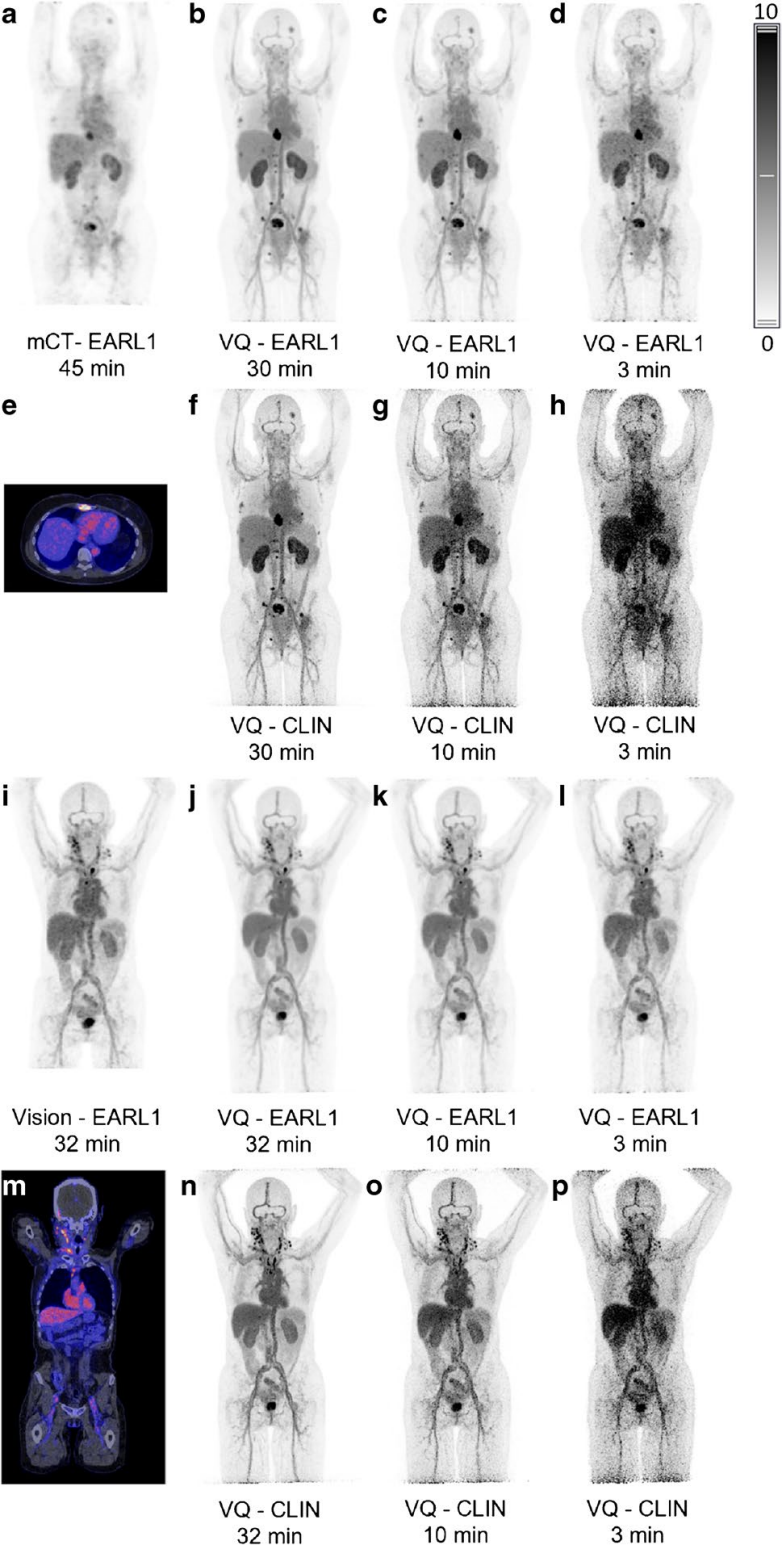# First-time imaging of [^89^Zr]trastuzumab in breast cancer using a long axial field-of-view PET/CT scanner

**DOI:** 10.1007/s00259-022-05777-x

**Published:** 2022-04-01

**Authors:** Adrienne H. Brouwers, Joyce van Sluis, Johannes H. van Snick, Carolina P. Schröder, Inge O. Baas, Ronald Boellaard, Andor W. J. M. Glaudemans, Ronald J. H. Borra, Adriaan A. Lammertsma, Rudi A. J. O. Dierckx, Charalampos Tsoumpas

**Affiliations:** 1 University of Groningen, University Medical Center Groningen, Medical Imaging Center, Department of Nuclear Medicine and Molecular Imaging, PO Box 30001, 9700 RB Groningen, The Netherlands; 2 Netherlands Cancer Institute, Department of Medical Oncology, Amsterdam, The Netherlands; 3 University of Utrecht, University Medical Center Utrecht, Department of Medical Oncology, Utrecht, The Netherlands; 4 VU Amsterdam, Amsterdam UMC - Location VU University Medical Center, Department of Radiology and Nuclear Medicine, Amsterdam, The Netherlands

Long axial field-of-view (LAFOV) PET/CT scanners have been introduced recently [1, 2], which offer numerous advantages [3]. One important advantage of using LAFOV PET/CT for imaging ^89^Zr-labelled monoclonal antibodies (mAbs), i.e., immunoPET, is the substantial increase in sensitivity compared with standard axial field-of-view (SAFOV) PET/CT systems, which may lead to a remarkable image quality improvement. This first study showcases such improvement for immunoPET with Biograph Vision Quadra™ (VQ) LAFOV PET/CT (Siemens Healthineers, Knoxville, TN, USA).

Two patients suffering from metastatic HER2-positive breast cancer were administered with 37 MBq [^89^Zr]trastuzumab in order to assist clinical decision-making [4, 5]. Patients were scanned 4 days postinjection with a Biograph™ mCT PET/CT (patient A) or a Biograph Vision™ PET/CT (patient B) (Siemens Healthineers, Knoxville, TN, USA), according to local standard operating procedures with overall scan durations of 45 min and 32 min, for, respectively, mCT and Vision. Following the clinical scans, patients were scanned with VQ. For VQ, we choose to apply a long scan duration of 30 min (patient A) and 32 min (patient B) to improve image quality rather than shortening the overall scan duration, as compared to Vision. For SAFOV systems, the acquisition and reconstruction parameters complied with EARL1, whilst for LAFOV, we also applied clinically (CLIN) recommended settings (Table 1) [6, 7].

**Table 1:** Acquisition and reconstruction parameters for the different systems

PET/CT system	Acquisition method	Reconstruction protocol name	Reconstruction settings
Biograph mCT	Step and shoot: 5 min per bed position (bp), 9 bp	EARL1	OSEM, 3i21s, size 256 × 256 × 488, voxel size 3.2 × 3.2 × 2.0 mm^3^, 6.5 mm FWHM Gaussian filter
Biograph Vision	Flow: continuous bed motion, 8 min per body pass, 4 passes	EARL1	OSEM, 4i5s, size 220 × 220 × 706, voxel size 3.3 × 3.3 × 1.5 mm^3^, 7 mm FWHM Gaussian filter
Biograph Vision Quadra	Single bp	EARL1	OSEM, 4i5s, size 220 × 220 × 708, voxel size 3.3 × 3.3 × 1.5 mm^3^, 7 mm FWHM Gaussian filter
	Single bp	CLIN	OSEM, 4i5s, size 440 × 440 × 708, voxel size 1.6 × 1.6 × 1.5 mm^3^, no filtering

*EARL* European Association of Nuclear Medicine Research Ltd., *OSEM* 3D ordered subset expectation maximization with time-of-flight and point spread function, *i* iterations, *s* subsets, *FWHM* full width at half maximum

PET/CT images of patient A are shown in the top two rows (a-h), for patient B in the bottom rows (i-p). The same intensity scale, SUV range 0–10, applies for all images, except the fused images (e, m). Additional reconstructions of the Vision Quadra data were obtained, mimicking 3-min (d, h, l, p) and 10-min (c, g, k, o) acquisitions, illustrating more pragmatic scan durations.

As can be appreciated from these first human immunoPET images on a LAFOV system, the image quality improvement (f) is most spectacular when compared with the mCT (a). For example, in patient A, an additional small bone lesion was visualized with VQ in the pelvic area (f), which was not visible with the SAFOV system (a). Even when compared to the Vision (i), the VQ image (n) shows improved quality without applying any filter after reconstruction. Moreover, this image quality was improved even in the 10 min image compared with the 30–45 min acquisition needed for SAFOV systems.

Thus, this image shows that the large axial FOV system provides substantial improvement in image quality when applying currently preferred overall scan durations on SAFOV systems (45 min for mCT, 32 min for Vision). Additionally, with the new LAFOV system, there is room for further reduction of the overall scan duration with still very acceptable image quality, even for ^89^Zr-labelled mAb PET/CT studies.